# Effectiveness of Sliding Mechanics on a Hybrid Round-Rectangular Archwire for Maxillary Canine Retraction, Angulation Control, Root Resorption, Pain, and Patient Satisfaction in Adult Class II Division 1 Patients: A Preliminary Pilot Study

**DOI:** 10.7759/cureus.90101

**Published:** 2025-08-14

**Authors:** Mohammad N. Kheshfeh, Mohammad Y. Hajeer, Ahmad S. Burhan, Mhd Firas Al Hinnawi, Mowaffak A. Ajaj, Alaa Oudah Ali Almusawi, Ahmad Salim Zakaria

**Affiliations:** 1 Department of Orthodontics, Faculty of Dentistry, University of Damascus, Damascus, SYR; 2 Department of Biomedical Engineering, Faculty of Electrical and Mechanical Engineering, University of Damascus, Damascus, SYR; 3 Department of Orthodontics, Faculty of Dentistry, University of Al-Knooz, Basrah, IRQ; 4 Department of Orthodontics, School of Dental Sciences, University Sains Malaysia, Kelantan, MYS

**Keywords:** acceleration of orthodontic tooth movement, canine angulation, friction technique, orthodontic tooth movement, tooth movement techniques

## Abstract

Background and objective: Optimizing orthodontic tooth movement while minimizing complications remains a primary goal for both clinicians and patients. This preliminary study evaluated canine retraction using the sliding technique on a round cross-section archwire, employing a hybrid archwire designed to enhance movement efficiency while controlling canine angulation during retraction. This hybrid design combines a rectangular anterior segment for incisor control with a round posterior segment to reduce friction during canine retraction, differing from conventional single-section wires.

Methods: This study was conducted at the University of Damascus from October 2022 to May 2023 with six patients diagnosed with Class II, Division I malocclusion (mean age: 21.18 ± 2.16 years). After premolar extractions, evaluation focused on the retraction of the right and left canines. A custom hybrid archwire was used, featuring a 0.021 × 0.025-inch rectangular section for the incisor brackets and a 0.020-inch round section for the canine, premolar brackets, and molar tubes. The study assessed the rate of retraction, angulation changes, root resorption, and patient-related outcome measures (PROMs) until a Class I relationship was achieved. Model casts were digitally scanned and analyzed in 3D using Blender® 4.4 to quantify movement, standard lateral cephalometric images (T1 and T2) were obtained to assess angulation changes, panoramic radiographs (T1 and T2) were used to evaluate root resorption, and questionnaires using VAS were administered for PROMs.

Results: The overall mean retraction rate was 1.28 ± 0.13 mm/month. The mean canine retraction rates were 1.46 ± 0.13, 1.42 ± 0.16, and 1.43 ± 0.16 mm/month in the first, second, and third months, respectively. The average retraction time was 4.38 ± 0.75 months, the mean canine angulation change was 5.05 ± 2.03 degrees, the mean canine root resorption was 1.01 ± 0.44 mm, and patient satisfaction was high, with an average score of 93.67.

Conclusion: Sliding on a 0.020-inch round cross-section archwire can significantly increase the retraction speed of the upper canines while minimizing changes in canine angulation during retraction, with minimal resorption and high satisfaction. However, further validation is needed to confirm its clinical efficacy.

## Introduction

During the treatment of Class II cases in adults using camouflage techniques, the first premolars are commonly extracted [1]. This process involves retracting the canines, followed by the incisors, based on a one- or two-step retraction philosophy [2]. Such treatment can extend up to 36 months, posing significant challenges [3]. Prolonged orthodontic therapies often result in complications such as tooth decay and periodontal disease, creating a strong demand for expedited processing [4,5].

To accelerate orthodontic tooth movement, researchers have explored both surgical and non-surgical methods [6-8]. Surgical techniques, including corticotomy [9], piezocision [10], flapless cortico-alveolar perforations [9], and periodontal accelerated osteogenic orthodontics [11], have demonstrated efficacy over the past two decades. Non-surgical methods have also gained interest, addressing patient and clinician reluctance toward surgical interventions [12]. These approaches include physical methods (e.g., low-level laser therapy [13] and electrical currents [14,15]), pharmaceutical technologies (e.g., local injection of platelet-rich plasma [16] and vitamin D administration [17]), and mechanical techniques such as self-ligation [18,19].

Clinicians face two primary challenges during canine retraction: a low retraction rate and complications related to angulation and rotation [20]. Frictional and frictionless methods have been evaluated to improve movement rates and minimize complications [21,22]. The traditional friction technique for tooth movement typically involves guiding the tooth along a thick, rectangular wire. This wire provides sufficient rigidity to maintain control during retraction and is designed to fit entirely within the slot [22]. However, the technique is known to substantially increase friction during sliding, thereby hindering movement [23]. Nonetheless, it is believed to minimize or prevent distal angulation of the tooth [22]. While rectangular wires were initially favored, their increased height led to higher frictional forces, significantly hindering tooth movement [23]. Sliding techniques using round cross-section archwires, designed to reduce friction with brackets, have emerged as a promising approach to accelerating tooth movement. Their design allows for maintaining a higher wire height, which in turn minimizes changes in canine angulation during retraction [22].

Although Huffman and Hamid clinically introduced this concept, their studies were criticized for short follow-up periods (ten and four weeks, respectively), incomplete investigation and data collection until the canine reached a Class I relationship, and other methodological shortcomings. In particular, the split-mouth design necessitated the use of different wires for the right and left sides, which were subsequently welded at the midline, a configuration that hinders the correction of the buccal-lingual inclination of the incisors during canine retraction [24,25].

To overcome these limitations, the research team at the Department of Orthodontics, Damascus University, conducted extensive experiments leading to the development of an innovative hybrid orthodontic archwire. This archwire features a posterior segment with a round cross-section, measuring 0.20 inches in diameter, designed to fit into the brackets of canines and premolars as well as the tubes of molars. The anterior segment is rectangular to engage the brackets of incisors securely. Unlike conventional round or rectangular wires, this hybrid archwire is designed to combine the low-friction benefits of a round wire with the angulation control of a rectangular wire, representing a novel approach to optimize canine retraction. Since its performance had not been evaluated in clinical settings, a preliminary exploratory pilot study was initiated to assess its ability to accelerate canine retraction and minimize the complication of distal angulation during retraction. The primary outcome was the rate of canine retraction, while the secondary outcomes included changes in canine angulation, root resorption, and patient-reported satisfaction, all of which directly reflected the study objectives.

## Materials and methods

Study design and setting

This preliminary clinical intervention study aimed to evaluate the effectiveness of maxillary canine retraction using the sliding technique with a round-section wire. The study was conducted at the Department of Orthodontics, Faculty of Dentistry, University of Damascus, Syria. The research protocol was approved by the Biomedical Research Ethics Committee of the University of Damascus (Approval No.: DN-30082022-9) before the commencement of the study. Following university regulations, this study's classification as a pilot study without a control group did not require registration with clinical trial registries. The research was funded by the University of Damascus (Reference No.: 501100020595).

Patient selection

Six patients diagnosed with Class II, Division 1 malocclusion were recruited from the patient records of the Department of Orthodontics at the Faculty of Dentistry, University of Damascus. This research was conducted between October 2022 and May 2023. The inclusion criteria were adult patients aged 17-28 years, diagnosed with skeletal Class II malocclusion, as indicated by an ANB angle between 4° and 7°, and dental Class II Division 1 malocclusion according to Angle’s classification, with a treatment plan involving the extraction of the maxillary first premolars. Exclusion criteria included overjet exceeding 7 mm, moderate to severe dental crowding, loss of one permanent upper arch tooth, prior orthodontic treatment, chronic health conditions or medication use, poor oral hygiene, and periodontal disease. Patients were informed about the study protocol, and written consent was obtained after careful explanation.

Treatment sequence

Anchorage Reinforcement

**Figure 1:**
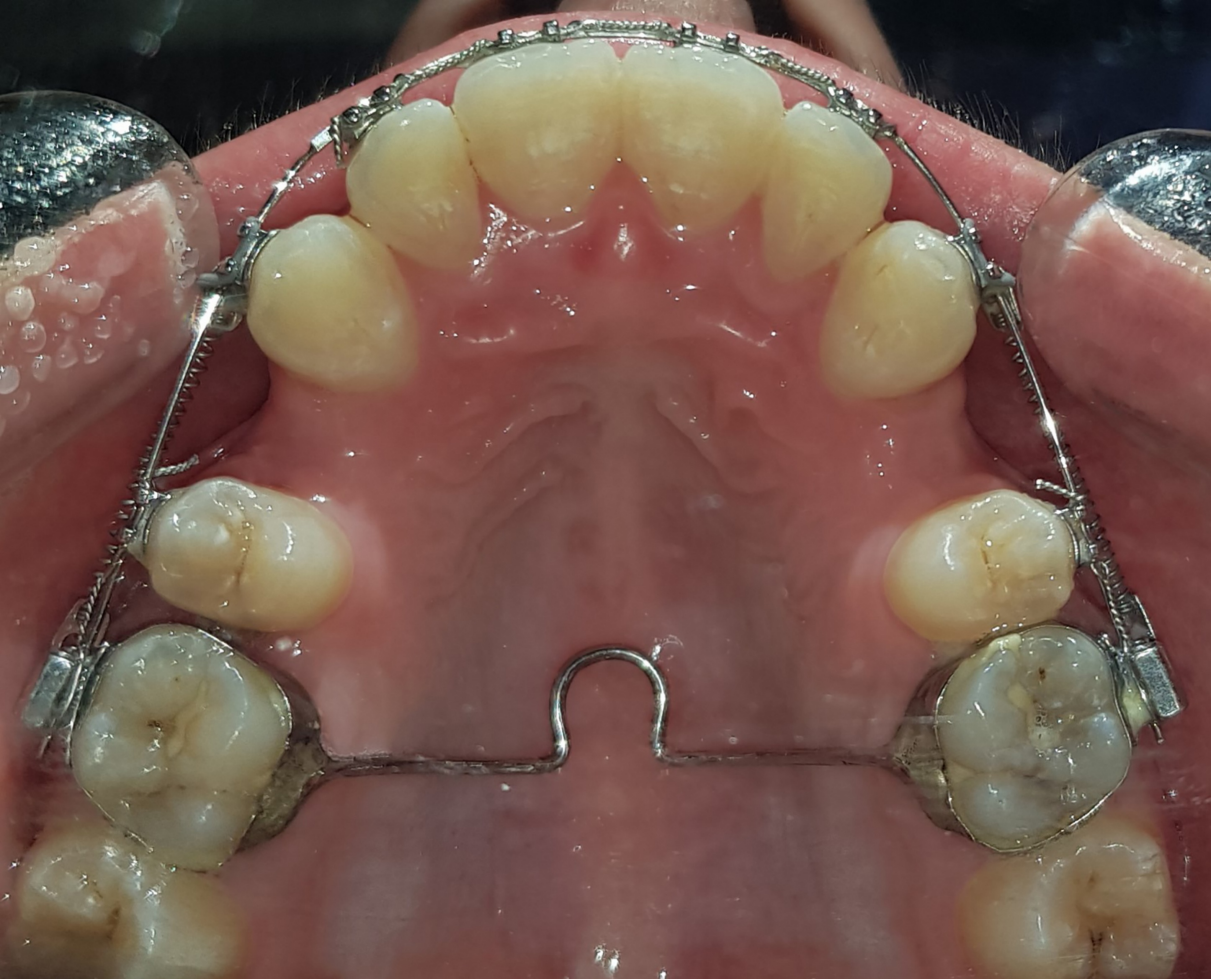
Occlusal view of orthodontic appliance with the transpalatal arch as an anchorage source.

Anchorage was reinforced with a transpalatal arch. Since our cases required only mild to moderate anchorage and canine retraction was carried out in a two-step technique, a maximum-anchorage appliance was not necessary. Therefore, we were satisfied with adding the transverse arch of the palatal vault, which provided minimal anchorage (Figure 1) [26].

Leveling and Alignment

The study utilized Pinnacle™ MBT prescription brackets and pre-adjusted fixed orthodontic appliances (Pinnacle™, Ortho Technology, West Columbia, South Carolina). Archwires were systematically replaced every 21 days in the following sequence: 0.012-inch NiTi, 0.014-inch NiTi, 0.016-inch NiTi, 0.016 x 0.012-inch NiTi, 0.017 x 0.025-inch NiTi, 0.019 x 0.025-inch NiTi, and 0.021 x 0.025-inch NiTi wires [2]. The decision to replace the archwires every 21 days was based on both biomechanical and clinical considerations. In vitro studies have demonstrated that the active force delivered by nickel-titanium (NiTi) archwires gradually decreases over time, with a noticeable decline after approximately three weeks of continuous activation. Replacing the archwires at 21-day intervals ensured that the applied forces remained effective for optimal alignment and leveling. Clinically, this interval also provided an appropriate balance between biological response and treatment efficiency, allowing sufficient time for periodontal tissues to adapt while minimizing periods of force decay. Furthermore, the protocol incorporated flexibility: if an archwire had not reached neutrality at the 21-day follow-up, it was maintained for an additional cycle, thereby avoiding premature replacement. Overall, the 21-day interval was selected as a pragmatic compromise that maintained consistent biomechanical efficiency, respected biological limits, and ensured systematic progression through the archwire sequence.

Innovative Hybrid Archwire

**Figure 2:**
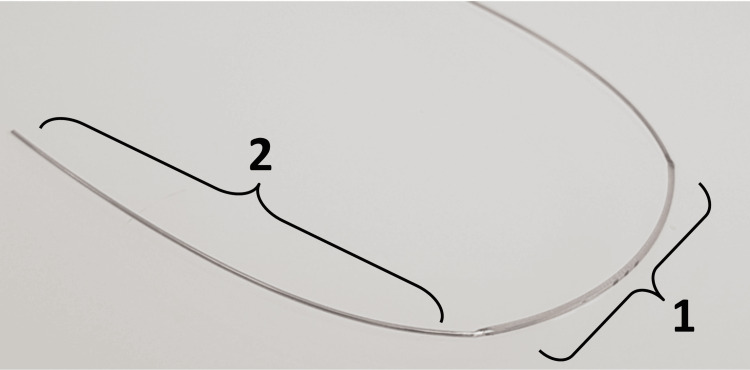
Hybrid archwire parts. 1: The rectangular cross-section of the archwire, which had the dimensions of 0.021 × 0.025 inch; 2: The round cross-section of the archwire, which had the dimension of 0.020 inch.

The hybrid archwire, developed by researchers M.N.K. and M.Y.H., was designed to achieve maximum stiffness within the canine and posterior teeth bracket slots while minimizing friction. For the anterior section (incisors), the wire dimensions were set to the maximum (0.021 x 0.025 mm) to ensure a tight fit. For the posterior section (canines extending to the wire’s end), a 0.020-inch stainless steel wire was selected based on a prior trial due to its low-friction characteristics (Figure 2). The sliding mechanism on this archwire has not been demonstrated to be effective for retracting anterior teeth.

Individual Wire Fabrication Method

The hybrid wire was individually fabricated by cutting a rectangular section from a 0.021 × 0.025-inch stainless steel archwire, corresponding to the span of the four incisor brackets, with an additional 2 mm on each side. Two round sections were cut from the remaining wire to form the posterior segments. The three wire pieces, that is, a rectangular anterior section and two posterior round sections, -were welded together using a neodymium-doped yttrium aluminum garnet (Nd:YAG) laser, creating a single, continuous hybrid archwire suitable for both en-masse and two-step retraction protocols.

The initial cutting and welding of the wire segments were performed in a certified external orthodontic laboratory. The research team made only minor final clinical adjustments when necessary. No images or videos were available from the laboratory phase. To enhance reproducibility, we provide a step-by-step written description of the fabrication and adjustment process, which can be reproduced in any orthodontic laboratory equipped with Nd:YAG laser welding.

The hybrid archwire was fabricated and clinically adjusted as follows: first, a 0.021″ × 0.025″ stainless steel rectangular segment was cut to span the four incisor brackets plus a 2 mm margin on each side, and two 0.020″ round stainless steel segments were cut to span from the canine bracket slots to the distal arch; all segments were ultrasonically cleaned and dried. The three pieces were then mounted on a typodont to verify passive fit anteriorly and smooth sliding posteriorly, with any minor bends trimmed to ensure precise alignment. Using an Nd:YAG laser welder under inert-gas shielding, each round segment was carefully fused to its corresponding end of the rectangular segment with controlled pulse energy and duration, optimized on test samples to achieve full fusion without thermal distortion, before polishing and inspecting each junction under a stereomicroscope to remove surface irregularities. The completed hybrid wire was re-engaged on the typodont to confirm a snug fit in the incisor brackets and minimal friction in the canine brackets; minor contour adjustments were made with precision pliers where binding occurred. After ultrasonic cleaning, the archwire was sterilized using a steam autoclave and then visually examined for defects. Intraorally, the wire was seated in the patient’s brackets, and if localized binding remained, gentle deflections were applied to the round segments with pliers to restore optimal sliding mechanics. All pre- and post-weld dimensions, laser parameters (pulse energy, duration, gas flow), and any intraoral adjustments were documented in a quality assurance log to ensure full reproducibility and traceability.

Extraction of First Premolars

The upper first premolars were extracted before the placement of the 0.017 x 0.025-inch NiTi wire during the leveling and alignment phase.

Canine Retraction Using Sliding on the Hybrid Archwire

**Figure 3:**
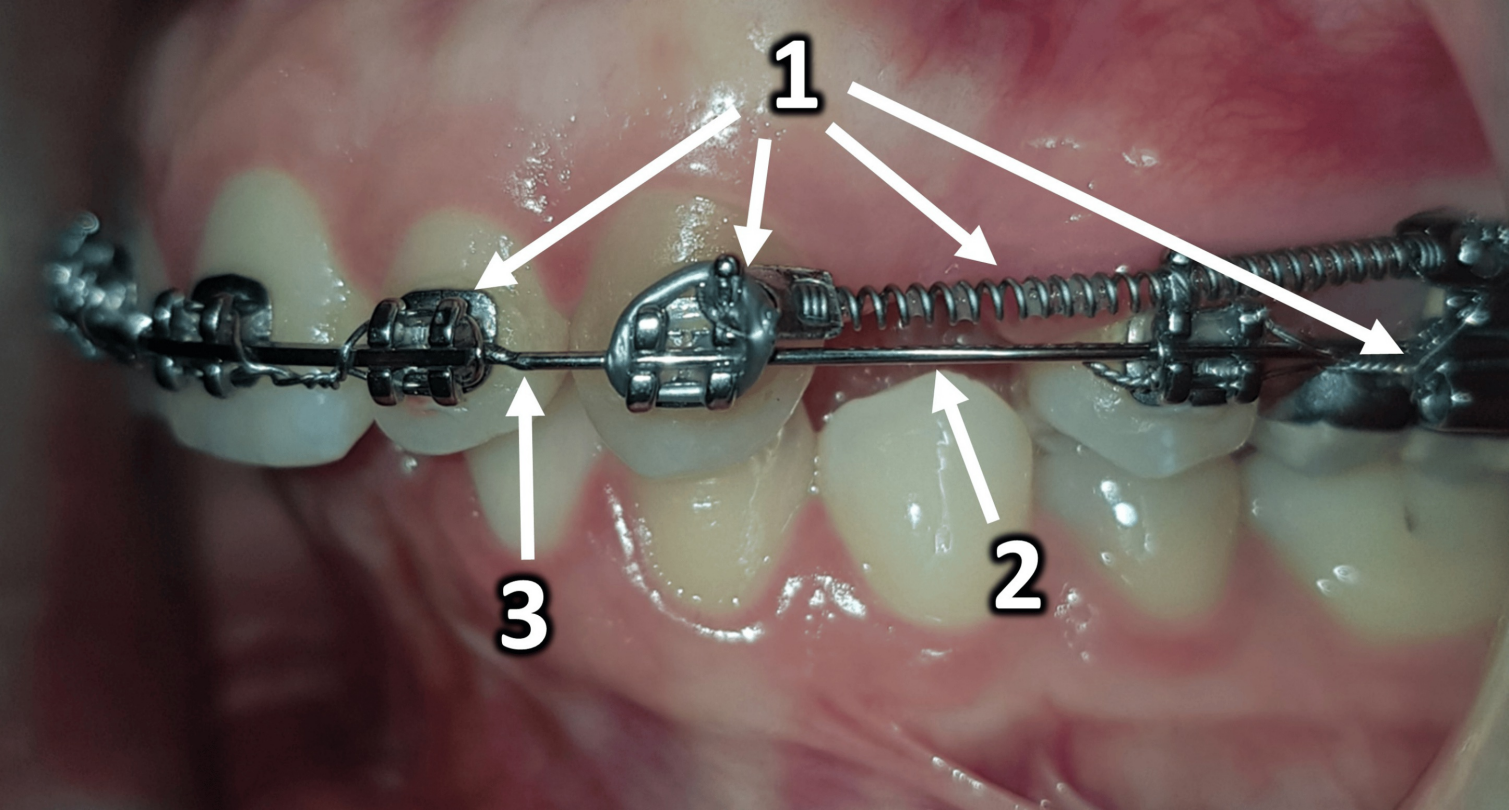
Lateral intraoral view of the orthodontic appliance in use. 1: Components of an orthodontic appliance; 2: the round cross-section archwire; 3: the welding junction.

A retraction force of 150 g was applied using a closed spring (NT3® closed coil, American Orthodontics, Sheboygan, Wisconsin). The spring extended from the maxillary canine bracket to the upper first molar hook, following the sliding technique on a round-section wire. The traction force was measured biweekly with an intraoral force meter (model 040-711-00, Dentaurum GmbH & Co. KG, Ispringen, Germany). The forces exerted by the springs were maintained at 150 g by measuring their output every two weeks. We used nickel-titanium springs, known for generating a consistent force over time; however, these springs were regularly inspected for any distortion or stress. Due to the potential for slight deformation of metal components, minor adjustments were made based on the measured force. If the force dropped below 150 g, the spring was reactivated or replaced; if it exceeded 150 g, the spring extension was adjusted or the spring was replaced accordingly. The hybrid wire consisted of a rectangular anterior section and a rounded posterior section (Figure 3). The intraoral force meter was calibrated before each clinical session according to the manufacturer’s instructions. Each spring was tested at two points during activation to ensure that the 150 g force was maintained. Springs were reactivated or replaced if deviations were detected.

Primary outcome measure: canine retraction rate

The movement of maxillary canines was assessed throughout the treatment period using study casts generated from alginate impressions. Impressions were taken at specific intervals: T0 (after the leveling and alignment phase), T1 (one month after initiating canine retraction), T2 (two months after initiating canine retraction), T3 (three months after initiating canine retraction), and TF (upon complete canine retraction, when a Class I canine relationship was achieved). These casts were scanned with an AutoScan DS-EX Pro (SHINING 3D, Hangzhou, Zhejiang, China) and measured with Blender® 4.4 (Blender Foundation, Amsterdam, Netherlands).

**Figure 4:**
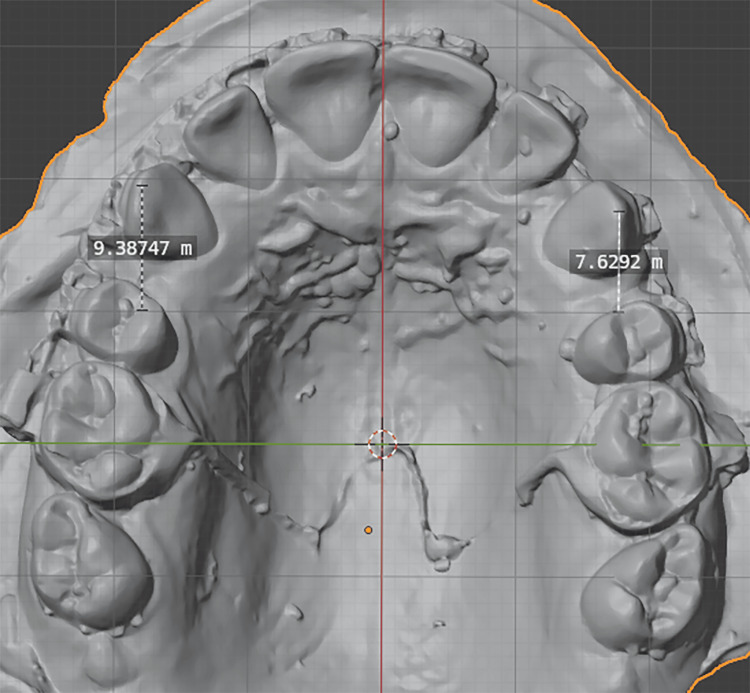
A screenshot of the Blender® 4.4 software that was used for taking measurements on 3D objects.

Canine retraction was quantified by projecting the apex of the maxillary canine and the medial end of the third palatal fold onto the maxillary midline, then measuring the distance between these points (Figure 4). For each time interval, the monthly retraction rate was calculated by dividing the measured distance by the corresponding time. The monthly average was determined from the twelve canines across the six patients. The overall retraction rate was calculated by dividing the total distance traveled by the total treatment duration (in months), which was four to five months in some cases.

Secondary outcome measure

Canine Angulation Change

**Figure 5:**
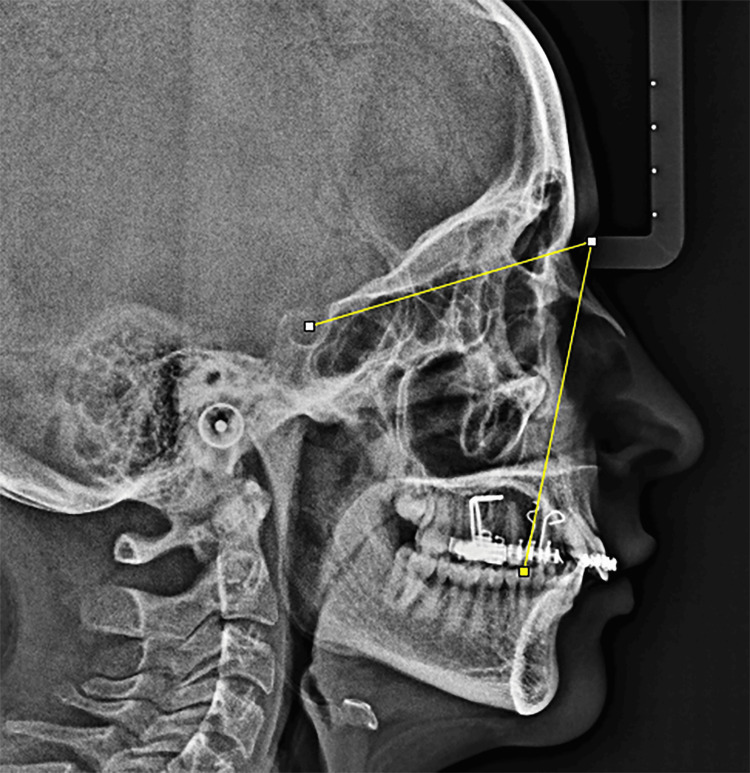
A screenshot of the ImageJ software when measuring the canine angulation on a lateral cephalometric image.

The change in canine angulation was analyzed at the start of the retraction process (T0) and upon completion (TF) using lateral cephalometric radiographs. Metal markers were placed on the right and left canines to track changes in canine angles as they reached a Class I canine relationship. This measurement was performed using ImageJ software (National Institutes of Health (NIH), Bethesda, Maryland; Figure 5). 

This outcome employed a two-dimensional cephalometric technique, which demonstrated acceptable reliability by minimizing errors from head positioning, magnification, and overlapping anatomical structures. Standardized cephalometric radiographs were obtained at both T1 and T2 using a digital computed radiography system (Cranexd Model PP1, Finland) on a single radiograph, adhering to rigorous standardized criteria. To reduce variability in radiographic analysis, all patients were positioned with the Frankfort horizontal plane parallel to the floor using a cephalostat, with a constant source-to-object distance. Metallic reference markers were used to verify alignment and monitor potential magnification variations between T0 and TF.

To assess changes in canine angulation individually, metal markers were affixed to both canines. The right canine was marked with a circular end, and the left with a triangular end. These locators, made from 0.021 × 0.025 mm stainless steel wire with a 12 mm apical extension, were positioned perpendicular to the bracket slot and parallel to the canine bracket hook, aligning with the canine’s longitudinal axis. Angulation was then determined by measuring the angle between each locator and the anterior cranial base (S-N reference plane). Angulation was defined as the distal crown tip, the angle between the long axis of the clinical crown and the Sella-Nasion (SN) plane, rather than root inclination. This method has been successfully applied in several studies, including those by Sueri and Turk [27].

Canine Root Resorption

Panoramic radiographs were obtained at the start (T0) and upon completion (TF) of the canine retraction process. At T0, the baseline length of the upper canine roots was recorded. At TF, changes in root length were measured to determine external apical root resorption (EARR) and the percentage of resorption. This analysis followed the technique described by Linge and Linge [28] and has been employed in several studies, including Jacobs et al. [29]. All measurements were performed using ImageJ software (National Institutes of Health, Bethesda, Maryland).

Pain, Discomfort, Functional Impairments, and Satisfaction

Questionnaire 1: The first questionnaire was given to all patients to assess their perceptions of pain, discomfort, and difficulties with chewing, swallowing, and speech at specific intervals: 24 hours (T1), 3 days (T2), and 7 days (T3) following the initiation of maxillary canine retraction with closed coil tension springs. The same questionnaire was repeated in the second month at 24 hours (T4), 3 days (T5), and 7 days (T6) after reactivating the coil tension springs, and again in the third month at the same intervals (T7, T8, T9) following the reactivation session. The questionnaire included six items: pain level, discomfort level, swallowing difficulty, chewing difficulty, speaking difficulty, and the need for analgesics (Appendix). This questionnaire has been used in several published papers in the field of accelerated orthodontics [30]. Responses were collected using a visual analog scale (VAS) for items 1 to 5, while item 6 was a binary (yes/no) question. The severity of each variable, based on VAS scores, was categorized as follows: mild (<20), mild to moderate (20 to <40), moderate (40 to <60), moderate to severe (60 to <80), and severe (80 to 100) [30].

Questionnaire 2: At the end of the third month of canine retraction, this questionnaire was administered to all patients. It included questions on the following items: (1) satisfaction with orthodontic treatment and (2) willingness to recommend this procedure to a friend. Responses to the first question were recorded using a VAS, whereas the second question required binary yes/no answers.

The questionnaire items were presented to patients in clear and understandable language. The researcher addressed all patient inquiries regarding the questionnaire without influencing their responses. Individuals reporting moderate to severe pain were instructed to complete the pain questionnaire before taking any analgesic medications. When necessary, they were permitted to take 500 mg of paracetamol (acetaminophen) to manage the pain.

Method error and assessor reliability

To assess measurement reliability, the principal researcher (M.N.K.) performed all measurements on a randomly selected subset of 10 digital scans and radiographs. The measurements were repeated after a two-week interval. Method error was quantified using Dahlberg’s formula, and intra-examiner consistency was assessed using intraclass correlation coefficients (ICC). Paired t-tests were conducted to detect any systematic bias between repeated measurements. All statistical tests were two-tailed, with a significance threshold of α = 0.05.

Statistical analysis

Statistical analyses were performed using IBM SPSS Statistics for Windows, Version 22 (Released 2013; IBM Corp., Armonk, New York). The mean right and left canine movement at each time point was calculated to determine the average monthly canine movement for each patient. The mean, median, minimum, and maximum monthly canine movement rates were then calculated for all patients at each time point. Finally, the overall canine retraction rate was determined by calculating the mean. The average angulation change and root resorption of the right and left canines were calculated, and the minimum and maximum values of the angulation change were identified.

## Results

Error of the method and reliability of the assessor

Dahlberg's errors were minimal (0.030-0.707 mm), indicating low random variability, and paired t-tests showed no significant systematic bias for all measurements (p > 0.05). ICC values were excellent across the board (0.982-1.000), confirming outstanding intra-assessor reliability. These findings demonstrate that our measurement protocol is both precise and reproducible.

Baseline sample characteristics

Six participants, with a mean age of 21.18 ± 2.16 years, were included in this study. Their data were analyzed following the completion of the follow-up period. Table 1 summarizes the patients' pre-treatment characteristics.

**Table 1:** Baseline characteristics of the sample at the beginning of the treatment.

Variables	Values
Number of patients	6
Gender (male/female)	2/4
Age (years) ± SD	21.18 ± 2.16 years
Pre-retraction extraction space	6.58 ± 0.37 mm

Rate of canine retraction, canine angulation, and root resorption

The average retraction space measured 5.56 ± 0.87 mm, with an average retraction time of 4.38 ± 0.75 months. During the first month of follow-up, the mean canine retraction rate was 1.46 ± 0.13 mm/month (Table 2). This rate remained relatively stable in the subsequent months, at 1.42 ± 0.16 mm/month during the second month and 1.43 ± 0.16 mm/month in the third. Over the entire treatment period, the overall average retraction rate was 1.28 ± 0.13 mm/month. By the end of the follow-up period, the mean canine angulation was 5.05 ± 2.03°. The proportion of canine angulation change per traveled distance was 0.89° ± 0.28°/mm, while the monthly angulation rate was 1.17° ± 0.5°/month. By the end of the follow-up period, the mean EARR was 1.01 ± 0.44 mm. The Resorption Percentage was 5.06 ± 1.98 %.

**Table 2:** Descriptive statistics of the upper canine retraction rate, angulation change, and root resorption. SD: standard deviation, N: number of patients, Max: maximum value, Min: minimum value, mm: millimeters. EARR: external apical root resorption.

Variables	Intervals	Mean ± SD	Median	Max	Min
Retraction space	T0-TF	5.56 ± 0.87 mm	5.55	6.84	4.55
Retraction time	T0-TF	4.38 ± 0.75 months	4.38	5.5	3.5
Canine retraction rate (N = 6)	T0-T1 (1^st ^month)	1.46 ± 0.13mm	1.41	1.65	1.31
	T1-T2 (2^nd ^month)	1.42 ± 0.16 mm	1.45	1.64	1.13
	T2-T3 (3^rd ^month)	1.43 ± 0.16 mm	1.39	1.7	1.25
	T0-TF	1.28 ± 0.13 mm/month	1.28	1.51	1.09
Canine angulation	T0-TF	5.05° ± 2.03°	4.51°	8.65°	2.93°
	Canine angulation rate	1.17° ± 0.5°/month	1.04°	2.16°	0.78°
	Canine angulation per distance traveled.	0.89° ± 0.28°/mm	0.83°	1.43°	0.61°
Canine root resorption	EARR	1.01 ± 0.44 mm	1.01 mm	1.63 mm	0.41 mm
	Resorption Percentage	5.06 ± 1.98 %	5.15 %	7.37 %	1.81%

Patient-reported outcome measures

On the first day following the retraction coil spring application in the first month (T1) and the day after spring activation in the second month (T4), pain intensity was reported as mild to moderate. At all other time points, only mild pain was observed. Discomfort was rated as mild to moderate only on the first day following the application of the retraction springs in the initial month (T1) and remained mild at all other times (Table 3). Difficulty chewing was mild to moderate during the first three days of the first month and the first day of the second month only (T1, T2, and T4), and mild during the remaining assessment times. Swallowing and speech difficulty were mild at all assessment times. Analgesic consumption was minimal, with only one patient requiring paracetamol (500 mg) at three distinct time points (T1, T4, and T8). All participants indicated that they would recommend the treatment to their friends and were willing to undergo the same procedure again. Overall, patient satisfaction was high, with an average score of 93.67.

**Table 3:** Statistical summary of patient-reported levels of pain, discomfort, swallowing difficulty, chewing difficulty, speech difficulty, and treatment satisfaction.

Variable	Time point	Mean (n=6)	SD	Median
Pain	T1	30	9.19	32
	T2	18.17	6.56	18
	T3	7.33	3.67	8.5
	T4	21	9.36	20
	T5	11.67	7.53	10
	T6	1.67	4.08	0
	T7	8	10.68	4.5
	T8	5.83	7.05	4
	T9	1.83	3.25	0
Discomfort	T1	26.67	12.12	28.5
	T2	20	14.14	20
	T3	15	13.78	10
	T4	15	16.43	10
	T5	11.67	11.69	10
	T6	6.67	10.33	0
	T7	8.33	7.53	10
	T8	6.67	10.33	0
	T9	5	8.37	0
Swallowing difficulty	T1	10.17	9.13	11.5
	T2	6.67	5.16	10
	T3	5	5.48	5
	T4	1.67	4.08	0
	T5	1.67	4.08	0
	T6	0	0	0
	T7	0	0	0
	T8	1.33	3.27	0
	T9	0	0	0
Chewing difficulty	T1	31.67	12.72	33.5
	T2	20	14.14	20
	T3	15	12.25	10
	T4	21.67	14.72	25
	T5	18.33	14.72	25
	T6	13.33	13.66	10
	T7	15	13.78	15
	T8	10	8.94	10
	T9	6.5	7.69	4
Speech difficulty	T1	4.5	5.43	2.5
	T2	1.67	4.08	0
	T3	1.33	3.27	0
	T4	0.83	2.04	0
	T5	0	0	0
	T6	0	0	0
	T7	0	0	0
	T8	0	0	0
	T9	0	0	0
Treatment Satisfaction		93.67	6.65	94

## Discussion

This preliminary study aimed to assess the efficacy of a round-shaped archwire sliding technique in expediting orthodontic treatment while minimizing canine angulation during retraction. To our knowledge, this study is the first to evaluate the effectiveness of maxillary canine retraction to achieve Class I relationships using the sliding technique with a 0.020-inch round-section archwire. A custom-designed archwire was individually manufactured for each patient to meet their unique needs. To optimize the tooth movement rate and activate the regional acceleration phenomenon (RAP), the maxillary first premolars were extracted during the leveling phase, allowing for sufficient healing of extraction sockets over a minimum two-month period before initiating canine retraction [31].

The focus on canine retraction, rather than en masse retraction, was driven by several considerations. First, testing the new technique in a controlled environment was crucial for accurately evaluating its impact on maxillary canine retraction [26]. Second, canine retraction involves fewer teeth and simpler movement patterns compared to en masse retraction, allowing for a more precise assessment [31]. The transpalatal arch played a pivotal role in these cases by maintaining transverse dimensions and assisting in molar de-rotation when necessary [32]. This strategic approach was integral to achieving stable and successful orthodontic outcomes [33].

The decision to use a hybrid archwire, despite the study's focus solely on canine retraction, was driven by the need to ensure an ethical treatment sequence. Using a purely round-section archwire could compromise the buccal-lingual incisor inclination correction, necessitating additional correction of the incisor angulation after canine retraction and before starting incisor retraction. This extra step would considerably extend the overall treatment time compared to initiating incisor retraction without delay. A similar philosophy to the hybrid-wire approach for incisor or en masse retraction is the bi-dimensional bracket system, in which anterior brackets feature a 0.018-inch slot and posterior brackets a 0.022-inch slot, with retraction carried out on a 0.018 × 0.025-inch archwire.

Orthodontic canine retraction has been extensively explored in the literature, with various methodologies including sliding and frictionless techniques, each offering multiple approaches [21,22]. In this study, the sliding technique was chosen for its advantages in improving esthetics [32] and enhancing patient comfort [34]. A preliminary clinical investigation was conducted to examine the impact of a round-section stainless steel archwire on the rate of maxillary canine retraction and the degree of canine angulation. The results were then benchmarked against findings from other studies on canine retraction.

The apparent increase in canine retraction may be related to the use of a hybrid archwire rather than natural variation. This interpretation is based on a comparison between our retraction rate (1.28 ± 0.13 mm/month for maxillary canines) and those reported in similarly designed pilot studies without control groups.

To evaluate the efficacy of the round-section wire in accelerating movement, we compared these results with studies that employed conventional sliding techniques, specifically those using a 0.019 × 0.025-inch stainless steel wire with standard brackets and a 150-g coil spring, without any acceleration method. For instance, Tiwari et al. reported a retraction rate of 0.83 ± 0.25 mm/month [35], Oz et al. observed a rate of 0.95 ± 0.31 mm/month [27], and Hussain and Sundari documented a rate of 0.82 ± 0.39 mm/month [36]. Remarkably, the round-section stainless steel wire increased the retraction rate by 0.26 to 0.44 mm/month, representing an acceleration of 27% to 46% under the same force conditions.

The mean maxillary canine angulation during retraction in this study was 0.89° ± 0.28° per millimeter. To assess the effectiveness of wire size in reducing angulation, these results were compared with studies that utilized sliding mechanics with smaller-diameter round wires during canine retraction. In comparison, Ziegler et al.’s study, which investigated sliding mechanics on a 0.018-inch wire, reported a mean angulation of 1.41° ± 0.89° per millimeter, indicating a significant reduction of approximately 37% in angulation in the present study [37].

Furthermore, the canines in this study exhibited an average monthly angulation change of 1.17° ± 0.5°. In contrast, Hayashi’s study, which employed sliding mechanics with a 0.018-inch diameter wire, reported an average angulation change of 7.94° over two months, corresponding to approximately 3.97° per month [38]. This comparison reveals a significant difference in angulation change between the two methods, suggesting that the technique employed in the current study achieves a more controlled retraction, resulting in approximately a 71% reduction in angulation change. Monthly monitoring of angulation changes would be ideal. However, due to ethical concerns regarding repeated radiation exposure, patients were monitored only once, after the retraction period had elapsed. In this study, the mean root resorption was 1.01 ± 0.44 mm, a clinically acceptable value that aligns well with previous reports, including those by Jacobs et al. [29].

All patients experienced only mild pain, discomfort, and functional limitations, and they expressed high overall satisfaction with the treatment. Moreover, every patient indicated they would recommend the procedure to their peers. This favorable response appears to be driven by the straightforward nature of the procedure, its rapid results, and the minimal, easily manageable pain and discomfort that did not interfere with daily social activities.

Although power calculations are often used to improve the accuracy of statistical analyses, the exploratory nature and small sample size of this pilot study preclude their meaningful application. The inherent instability of estimates in such a limited sample can lead to misleading power estimates that might erroneously characterize the study as underpowered. Importantly, the aim of a pilot study is not to produce definitive results, but to efficiently inform the design of larger, confirmatory experiments, making extensive power analysis impractical and misaligned with the study’s primary objectives.

Similarly, formal tests for outliers, such as the Grubbs test, are problematic in this context because each value in a small sample exerts a disproportionate influence on the arithmetic mean. These tests rely on assumptions that may not hold with a very limited number of observations, potentially misidentifying normal variation as outliers. This sensitivity highlights the importance of cautious interpretation of outlier analyses in pilot studies, ensuring that the conclusions drawn remain robust while acknowledging the exploratory nature of the research.

In light of these findings, we recommend conducting a prospective, two-arm randomized controlled trial to compare the novel hybrid archwire with conventional sliding mechanics using either 0.017 × 0.025-inch or 0.019 × 0.025-inch wires. The suggested sample size can be calculated using the current findings for a two-sample t-test. With a canine retraction rate of 0.95 mm/month (SD = 0.31), a minimal clinically significant difference of 0.30 mm/month (i.e., a 35% increase in speed), α = 0.05, and 85% power, 17 patients are required per arm. With an attrition rate smaller than 5%, the required sample size for each group is 18 patients. The trial should evaluate key outcomes, including the rate of tooth movement, changes in angulation, patient-centered measures (such as pain, comfort, and overall satisfaction), the extent of root resorption, and alterations in alveolar bone height. Such a study will provide robust evidence on the clinical advantages and potential limitations of the innovative hybrid archwire compared to traditional methods, ultimately informing future orthodontic treatment protocols. We further recommend longitudinal CBCT assessments at defined post‐treatment intervals to evaluate volumetric alveolar changes and ensure the durability of canine position over time.

Limitations of the current study

This pilot study is the first to investigate the use of a round-section archwire to accelerate maxillary canine retraction while minimizing changes in angulation, associated root resorption, and patient-related outcome measures. However, it is not without limitations. Notably, these include the absence of a control group and a relatively small sample size. Moreover, the exploratory design and pilot scale preclude robust statistical generalization, underscoring the need for adequately powered, two‐arm randomized controlled trials to confirm these preliminary findings. Future research should address these limitations and investigate potential long-term complications, such as changes in the level of alveolar bone supporting the canine.

In particular, the reliance on Nd:YAG laser welding and stereomicroscopic inspection for hybrid-wire fabrication may limit clinical accessibility; alternative joining methods or prefabricated systems should be explored to enhance practicality. Additionally, the short follow-up period and use of two-dimensional radiographs may underdetect volumetric bone changes and root resorption; incorporating CBCT in future protocols is recommended to improve measurement accuracy and monitor long-term stability.

## Conclusions

The current study demonstrates the promising potential of sliding mechanics on round-section archwires to enhance maxillary canine retraction. Given the exploratory nature of this pilot study, the findings should be interpreted with caution and are not intended to directly guide clinical decision-making at this stage. The small pilot sample and absence of a randomized control group in this preliminary investigation limit the statistical power and direct comparability of the findings. This approach significantly accelerated canine retraction and effectively reduced angulation changes, with minimal root resorption, while achieving high patient satisfaction. This improvement in overall treatment effectiveness may increase the feasibility and acceptance of this approach in contemporary orthodontic practice. Moreover, the relatively short follow-up period, confined to the retraction phase, does not permit an assessment of long-term stability or alveolar bone remodeling, underscoring the need for extended post-treatment observation. By simplifying procedures and yielding superior outcomes, this innovative method holds great promise for future orthodontic applications, pending further validation through controlled clinical trials and extensive research. Future studies should therefore enroll larger cohorts, include appropriate control arms, and employ longer-term and three-dimensional imaging protocols (e.g., CBCT) to corroborate treatment efficacy and assess volumetric tissue changes over time.

## Figures and Tables

**Figure 6:**
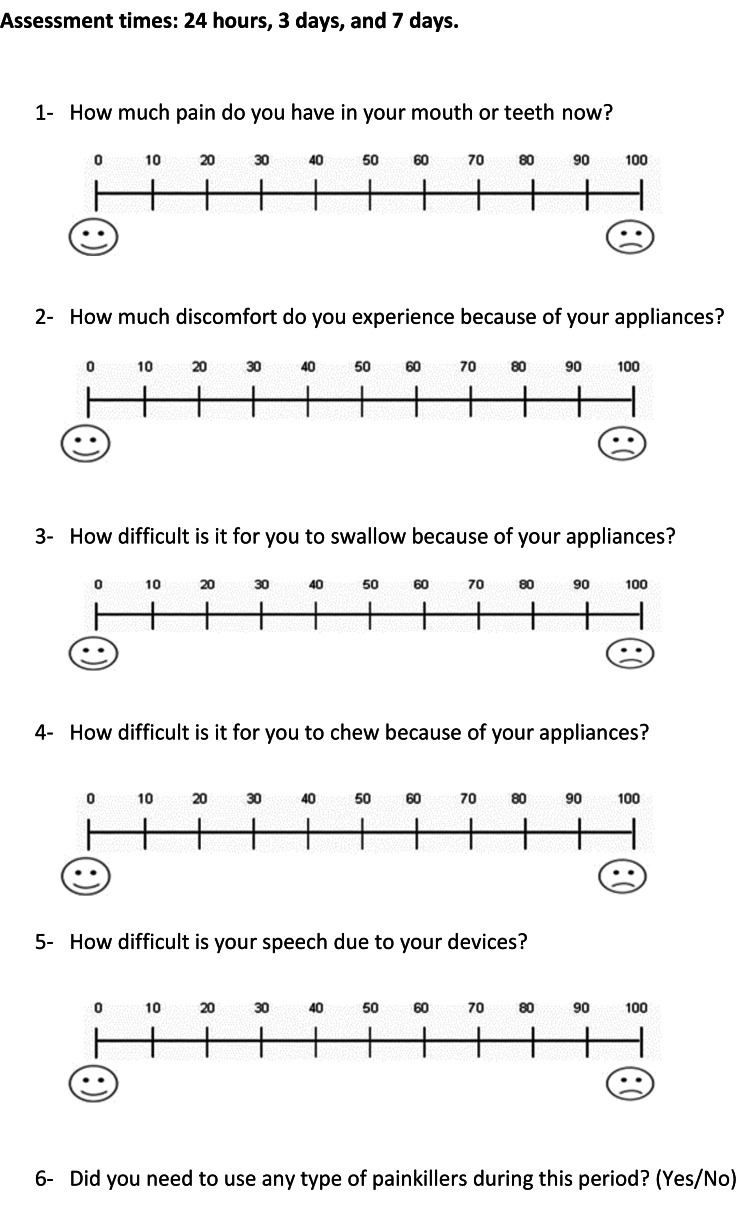
The questionnaire used in the current study. The first questionnaire was administered to all patients at the following intervals after the start of spring-applied retraction: 24 hours (T1), 3 days (T2), and 7 days (T3). It was then repeated in the first week of the second month at 24 hours (T4), 3 days (T5), and 7 days (T6) post-retraction. The last assessment was in the first week of the third month; it was administered again at 24 hours (T7), 3 days (T8), and 7 days (T9) post-retraction.
